# Trauma, poverty and mental health among Somali and Rwandese refugees living in an African refugee settlement – an epidemiological study

**DOI:** 10.1186/1752-1505-3-6

**Published:** 2009-05-26

**Authors:** Lamaro P Onyut, Frank Neuner, Verena Ertl, Elisabeth Schauer, Michael Odenwald, Thomas Elbert

**Affiliations:** 1 Mbarara University of Science and Technology, Uganda; 2 University of Konstanz, Germany; 3 University of Bielefeld, Germany; 4 Vivo International, Zur Setze 7, 78476 Allensbach, Germany

## Abstract

**Background:**

The aim of this study was to establish the prevalence of posttraumatic stress disorder (PTSD) and depression among Rwandese and Somali refugees resident in a Ugandan refugee settlement, as a measure of the mental health consequences of armed conflict, as well as to inform a subsequent mental health outreach program. The study population comprised a sample from 14400 (n = 519 Somali and n = 906 Rwandese) refugees resident in Nakivale refugee settlement in South Western Uganda during the year 2003.

**Methods:**

The Posttraumatic Diagnostic Scale (PDS) and the Hopkins Symptom Checklist 25 were used to screen for posttraumatic stress disorder and depression.

**Results:**

Thirty two percent of the Rwandese and 48.1% of the Somali refugees were found to suffer from PTSD. The Somalis refugees had a mean of 11.95 (SD = 6.17) separate traumatic event types while the Rwandese had 8.86 (SD = 5.05). The Somalis scored a mean sum score of 21.17 (SD = 16.19) on the PDS while the Rwandese had a mean sum score of 10.05 (SD = 9.7).

**Conclusion:**

Mental health consequences of conflict remain long after the events are over, and therefore mental health intervention is as urgent for post-conflict migrant populations as physical health and other emergency interventions. A mental health outreach program was initiated based on this study.

## Background

The firm establishment of Posttraumatic Stress Disorder (PTSD) as a category of mental ill health in the Diagnostic and Statistical Manual (DSM) has inspired fervent research into its epidemiological manifestations and characteristics.

Since the critically acclaimed National Co-morbidity Survey of 8,098 subjects in the United States [1]other epidemiological studies have established PTSD prevalence rates and other epidemiological characteristics in European [2-6], Australian and other western populations.

More recently, research has focused on post-conflict refugee populations from low-income countries who have relocated to western countries. These include Southeast Asian (Indochinese) [7], Kosovar [8], Cambodian [9], or Bosnian refugees [10] relocated to the United States or Australia [11], or to the United Kingdom [12], who in general show higher prevalence rates than western populations.

In a similar vein, emerging research in post-conflict populations relocated to other low-income host countries or remnant in their countries of origin, such as Bosnian refugees relocated to Croatia [13], Afghan refugees resident in Pakistan [14] or Tibetan refugees resident in India [15] continue to demonstrate the disturbingly high prevalence rates of traumatic stress reactions and related disorders among post-conflict populations.

This is especially true for Africa, where many of the world's conflicts, displacing thousands of survivors, take place. According to UNHCR, Africa hosts at least 20% and rising of the world's refugees and other migrant populations [16].

Studies carried out among post-conflict populations in Africa in order to quantify the incidence and prevalence of PTSD and depression are of growing interest. For example, De Jong et al. cite 37.4% PTSD prevalence in Algeria and 15.8% in Ethiopia in a study encompassing four different post-conflict settings with differing backgrounds [17].

In a representative survey conducted in Rwanda after the Rwanda genocide, Pham et al. found that 24.8% of the respondents met the symptom criteria for PTSD [18]. Dyregrov et al. found that 79% of the youth in Rwanda were at risk of developing PTSD [19]. In a later study, Schaal et al. found a 44% prevalence of PTSD among respondents who were children at the time of the genocide [20].

Uganda, a small East African country with a population of little over 28 million inhabitants, has long been a host to refugees from the region due to various conflicts. One of the bigger refugee populations has been the refugees from the Rwandan genocide in the year 1994. Other populations include the Somali refugees from the conflicts in Somalia dating to 1991. Adequate information about these refugees is necessary in order to plan appropriately for emergency care and mental health care provision.

Some studies have been conducted in Uganda on some refugee populations. For example, Neuner et al. found 50.5% PTSD prevalence among Sudanese refugees resident in northern Uganda, compared to 44.6% of Sudanese nationals still resident in the Sudan and 23.2% of Ugandan nationals resident in north Uganda [21,22]. To date, such data has been unavailable about the sizable Rwandese and Somali refugee populations in Uganda, mostly resident in the south of the country.

The concern that data on the prevalence of diagnosed common mental health disorders, including PTSD, among post-conflict populations in Africa is still scanty, is compounded by the fact that methodological inconsistencies still prevail in existing and continuing studies. For instance, most researchers use diagnostic instruments whose translations have not been validated in the target population. Setting the standard, Mollica et al. rightly validated the Havard Trauma Questionnaire among the Indochinese before using it for measurements within the same questionnaire [23]. Other studies, however, have used the same questionnaire without validating it within the target population, thus raising questions about the quality of measurements. Additionally, many researchers still merely estimate risk for PTSD, without affirming a PTSD diagnosis. Such prevalence outcomes are difficult to compare with studies where PTSD is diagnosed according to the DSM-IV.

Since more and more Africans are fleeing from conflicts in their own land to neighbouring low-income host nations, such as Rwandese and Somali refugees fleeing to Uganda, the urgency consists in not only learning more about the prevalence rates of PTSD and its co-morbid disorders among refugee and other displaced survivor populations with a view to planning mental health outreach to the populations of concern, but also to acquire this knowledge by employing studies that meet international methodological standards.

Our goal was a comprehensive methodologically stringent epidemiological study in order to establish rates of trauma exposure, and subsequent PTSD and depression prevalence among post-conflict refugee survivors in an African setting. Unlike any other previous studies, a careful measure of the socio-economic status of respondents was undertaken, in order to investigate how poverty interacts with mental health disorders in a post-conflict population resident in a low-income country. The bulk of interviews, carried out by local trained lay interviewers, only proceeded after the validation exercise, and even then only under close supervision.

## Methods

### Setting

Nakivale Refugee Settlement is one of the 8 official refugee camps in Uganda. It is situated in South-western Uganda 60 kilometres from Mbarara, the third largest town in Uganda. Nakivale settlement, 42 square kilometres in size, is also one of the oldest in Uganda, having already been in existence by 1952. At the time the study was carried out, (2003), Nakivale was host to 14,400 refugees -12,000 of them Rwandan Hutu refugees from the conflicts in the early 1990s – and slightly over 500 Somalis who fled to Uganda via Kenya [24]. A confirmatory age-restricted replica study was carried out in 2006 in connection with a human genome study [25].

These numbers are according to the official camp statistics from the camp administration. The refugees receive basic health care and a minimum of food aid. Educational opportunities are available for primary school-age children, and every family can supplement its income through agriculture from land granted by the Ugandan government at no cost. Mental health support for the refugees has been negligible.

Refugees are a protected population and refugee settlements are protected areas under the joint custodianship of UNHCR and the Ugandan government, represented in the settlement by the Camp Commandant.

Permission to carry out the study was obtained from both the above-named parties, and the study was approved by the ethical boards of Mbarara University of Science and Technology, Uganda and the University of Konstanz, Germany.

During the pre-inquiry phase of the study, the communities and their leaders were informed in depth about the proposed study, and the sampling rationale was explained in brief.

At the very outset, it was made clear to all the respondents that the interviews were entirely voluntary, and no monetary or food-item inducements would be offered.

### Participants

Participants came from the Rwandese and Somali refugees resident in Nakivale refugee settlement in South Western Uganda. The inclusion criteria encompassed all Rwandese (Hutu) and Somali refugees of either sex above the age of 12 officially registered and resident in Nakivale refugee settlement.

Participants were fully informed before participation, albeit verbally, since most of them were analphabetic. They gave a verbal informed consent before the interview was begun.

Since this study was completed, the respondent Somali refugee population has been resettled almost in entirety. It has been replaced by new refugees, whom the data here presented may not represent.

### Aims

We aimed to (a) assess the general nutritional, socio-economical, educational and physical health status of the refugees (b) assess the prevalence of mental disorders associated with exposure to stressful and traumatic armed conflict situations, specifically posttraumatic stress disorder and depression and (c) ascertain the types, descriptions and numbers of extremely stressful and traumatic events to which survivors were exposed.

It was expected that PTSD and depression could be identified in this non-western population; that the PTSD construct would prove valid in this population, and that prevalence rates would resemble those from studies based on other non-western post-conflict populations in low-income countries.

As already mentioned, the bulk of data for this study was collected in the year 2003. However, an age-restricted replica study was conducted in the year 2006, which largely confirms the results here presented. For purposes of clarity and brevity, these will be reported separately.

### Instrumentation

#### Socio-demographic interview

We employed a previously developed sociodemographic survey to assess nutritional, educational, socio-economic and physical health indicators as well as displacement and general demographic information [21,22,26].

The interview began with personal information like gender, age and marital status, as well as displacement history. Nutrition was assessed by asking for the number of meals eaten the previous day and by listing the various food items consumed. Since the refugees rarely have steady income flow, their economical status was ascertained by counting the number of essential household assets such as blankets, mattresses, cooking pots and water containers. These items would be acquired whenever any sort of income was available. In analysis, the value of the items was then weighted according to then-current market prices in Uganda. This value is presented as American dollars in the data. Educational achievement was indicated by the number of years of schooling completed. Physical health was evaluated against a checklist of common illnesses experienced within the last-one-month period. Such illnesses include malaria, cough, headache, tuberculosis, epilepsies, scabies, leprosy and sexually transmitted diseases.

#### Event Checklist

A 34-item Event Checklist developed by this group of researchers in previous studies with post-conflict populations was used to identify extremely stressful and traumatic events that the interviewees had experienced within their lifetimes [21,22,26]. The list includes different event types including combat, assaultive violence, torture, sexual violence, accident, natural disasters as well as forced circumcision and marriage. Each event was scored as *ever experienced *(within the lifetime) and *experienced in the past year*. The number of different experienced and witnessed types of traumatic events was used as an estimate of the severity of trauma exposure.

#### Assisted Self-Report

A key objective of this study was to evaluate the efficacy of local capacity building within a community-based approach both in the procedure of scientific inquiry, and in the provision of treatment. A local team of non-professional interviewers therefore conducted the interviews under supervision after rigorous training. In order to screen for Post Traumatic Stress Disorder (PTSD), the Posttraumatic Stress Diagnostic Survey (PDS) was employed as a standardized assisted self-report instrument [27]. Both the frequency and severity of PTSD were indicated.

For this study, the PDS was chosen as the chief diagnostic tool because of its confirmed psychometric properties as a self-report questionnaire [27,28]. It is the only self-report measure to assess all six (A-F) criteria for PTSD in the DSM-IV. Part 1 of the PDS is a 13-item checklist of potential traumatic events. Part 2 consists of eight items that help determine if an event meets the DSM-IV definition of Criterion A. Part 3 assesses the frequency over the past month of the 17 PTSD symptoms, using a 4-point scale ranging from 0 – Not at all or only one time to 3 – 5 or more times a week/almost always. Part 4 assesses the impact of symptoms on various aspects of social and occupational functioning.

An eight-point list of possible functioning deficits (which the respondent attributed to posttraumatic symptoms) was applied. This included 1) ability to engage in occupational activities (earn a living), 2) ability to engage in constructive activities within the household such as performing household chores, 3) ability to sustain healthy relationships with friends 4) ability to engage in hobbies, 5) ability to take part in instructional activities such as schooling, 6) ability to sustain healthy family relations, 7) general satisfaction with life and 8) overall functions in all areas of life. This is presented in the results as sum score of functioning deficits.

The PDS yields both a dichotomous diagnostic score and a cumulative symptom frequency score. An individual PTSD symptom is counted as present if the corresponding PDS item is endorsed as a 1 or higher.

In our validations of the PDS, over an interval of approximately two weeks, test-retest cum inter-rater reliability for symptom severity achieved a kappa of 0.74, for diagnostic agreement between the two administrations. The PDS had reasonable diagnostic utility against a PTSD diagnosis based on the CIDI, with a sensitivity of .85, a specificity of .84, an efficiency of .79. A validation report of all instruments here cited is reported in detail by Ertl et al. [29].

#### Validation Interview

The Composite International Diagnostic Interview (CIDI) [30] version 2.1 was chosen as the clinician-administered instrument, which would validate the PDS in its local language translation as a diagnostic tool. In the validation, a sample of the respondents interviewed by the local interviewers (who used the PDS) were re-interviewed by clinicians using the CIDI section K, within a two-week period.

The Hopkins Symptom Checklist 25 (HSCL-25) was chosen as an assisted self-report interview to indicate the possibility of co-morbid depression [31,32]. The respective CIDI section E was used for validation purposes. A more extensive investigation with other sections of the CIDI was considered impractical given personnel and other constraints.

The entire questionnaire (encompassing the socio-demographic interview, the Event Checklist as well as the PDS and HSCL-25 diagnostic interviews) was then translated into the local languages Somali and Kinyarwanda using several steps of translations, blind back translations and subsequent corrections by independent groups of translators. Details of training, translation and validation of the local language instruments are elsewhere described [33].

### Procedure

#### Sampling

The Somali population totalled approximately 500 persons. They were mostly refugees from the 1991–1992 civil war in Somalia, who have fled to Uganda via Kenya. In addition to the war events, many had flight events that had forced them to flee further than their initial destinations of refuge, e.g. Kenya. For this population, a complete sample was carried out, i.e. a hut-to-hut interviewing procedure for all the huts was effected. Every Somali refugee above the age of 12 in every household permanently resident in the camp was interviewed.

Of the 14.400 refugees in this settlement, 12.000 are Rwandese, of mostly Hutu ethnic origin. These are refugees from the ethnic conflicts in Rwanda in the early 1990s. For this population, a single-stage cluster sampling procedure was employed, with cluster units of unequal size (the households were of unequal size); the households being the listing units. The list of households in each zone constituted the sampling frame. From the lists of households in each Rwandese zone, a number of households were sampled at random in the ratio of the size of the zone in proportion to the total number of Rwandese households in the camp. Since the zones were arranged in no discernible order, the middle of the zone (usually a trading centre), was used as a starting point. A hut-to-hut interview procedure was enacted with huts being selected according to the random cluster sample.

The interviewers sampled huts outwards in the four directions from the centre of the village. All present household members of the selected huts were interviewed, beginning with the household head and including any adolescents above the age of 12. Every attempt was made to interview members of the specific huts before any re-assignments were made. In the sample, assignments were made without replacement. Each respondent was interviewed once by a local, trained non-professional interviewer, except for the random sub-sample that was re-interviewed within two weeks by expert clinicians using the CIDI for purposes of validation. Both the assisted self-report and expert interviews were face-to-face at-home interviews. The response rate was over 90%.

#### Validation

In order to validate the instruments that were translated, a validation exercise was carried out by the expert team. A random sample of the interviews that were conducted by the local trained lay interviewers using the PDS (n = 98) were re-interviewed by the expert clinicians using the CIDI Section K, within a time space of two-weeks. The validation was both a test-retest validation since the same patients were interviewed twice within a two-week period using two different instruments, as well as an inter-rater validation since the expert team re-tested the interviews done by the trained lay local interviewers.

The Kinyarwanda version of the PDS (n = 60; 6.5% of the Rwandese interviews) had a kappa score of 0.72, a sensitivity of 0.83 and a specificity of 0.89. The Somali version of the PDS (n = 38; 7% of the Somali interviews) had a kappa score of 0.71, a sensitivity of 0.88 and a specificity of 0.85. Both local instruments had a joint kappa score of 0.74, a sensitivity of 0.86 and a specificity of 0.88. Additionally, the correlation between the PTSD diagnosis made by the trained lay interviewers using the PDS and the diagnosis made by the expert clinicians using the CIDI was 0.732; p < .001.

The section of the HSCL-25 measuring depression was validated using the CIDI Section E within a two-week period. The kappa value of the Rwandese version of the HSCL-25 depression section where a cut-off score of 1.75 was employed was 0.11; where a cut-off score of 1.67 was employed was 0.24 and 0.46 using the Bolton algorithm. The Rwandese version of the HSCl-25 has a sensitivity value of 0.10 for a 1.75 cut-off score, 0.20 for a cut-off score of 1.67 and 0.50 for the Bolton algorithm. This version also had a specificity value of 0.98 at the 1.75 cut-off score, 0.98 at the 1.67 cut-off score and 0.93 when the Bolton algorithm was employed. (This algorithm was developed and tested by the Havard Program in Refugee Trauma. Since the HSCL-25 was created prior to the DSM Depression criteria, it is not entirely consistent with the DSM 'A' criteria for depression. The algorithm was developed to match HSCL-25 Depression questions to DSM Criteria for Major Depression [34]. The Somali version of the HSCL-25 depression section had a kappa value of 0.35 at the 1.75 cut-off score, 0.37 at the 1.67 cut-off score and 0.13 when the Bolton algorithm was employed. This version also had a sensitivity of 0.57 at the 1.75 cutoff score, 0.64 at the 1.67 cut-off score and 0.79 using the Bolton algorithm. A specificity of 0.77 was achieved at the 1.75 cut-off score, 0.73 at the 1.67 cut-off score and 0.36 employing the Bolton algorithm.

Taken together, both local language versions of the depression section of the HSCL-25 achieved a kappa value of 0.31 at the 1.75 cut-off score, 0.37 at the 1.67 cut-off score and 0.35 employing the Bolton algorithm. A joint sensitivity value of 0.38 at the 1.75 cut-off score was achieved, 0.46 at the 1.67 cut-off score and 0.67 employing the Bolton algorithm. A joint specificity value of 0.90 at the 1.75 cut-off score, 0.89 at the 1.67 cut-off score and 0.73 using the Bolton algorithm was achieved. The Event List used was a newly-arranged version of one used in a previous study [21]. It showed a high internal consistency (Cronbach's α > .88); significant retest-reliability (r = .73; p < .001) and significant accordance with the CIDI Event List. The Socio-Demographic Survey produced data which proved to be satisfactory. Of 36 items, 31 reached significance with correlations between r = .38, p = .021 and r = .97, p < .001; and kappa scores between κ = .48; p < .001 and K = 1.00, p < .001 respectively.

A more detailed account of the validation results can be consulted in Ertl et al. [29].

### Results

#### Demographic Profile of the Sample

Over 1491 interviews were completed, of which 1422 were used in the analyses (n = 516 Somalis and n = 906 Rwandese). The remaining interviews were excluded because the respondents were Kinyarwanda-speaking but were not ethnic Rwandese.

### Religion and Marital Status

All the Somalis except 1 were Muslims (n = 515) while 90.5% of the Rwandese were Christian. More than half of the respondents (57.9%) were married, 26.4% were single, and 8.5% were widowed while the remaining 6.8% were separated, co-habiting or divorced.

### Education and Occupation

Of the respondents, 34.6% had never been to school, while 46.3% had had basic primary education (1–7) years of schooling. 17% had had at least 12 years of schooling, which translates to secondary education. Only 1.5% had had more than 13 years of schooling, which translates to tertiary (professional, vocational or university) education. The mean number of years of schooling was 3.82 (SD = 3.83).

Before their first experience of displacement, 35.8% of the respondents were farmers, 29.5% had no occupation and 13.8% were displaced as students. At the time of the study, 41.6% of the respondents had no occupation, 39.5% were farmers and 8.2% worked within a household. Other occupations included working for non-governmental organizations, working for the police or army or operating a restaurant or repair shop. Differences between national groups in occupation before displacement were significant; χ^2 ^= 459.5, p < .000.

Only 2.9% of the Somalis claimed to have been farmers before displacement, and just 0.58% claimed to be farmers at the time of the study, in contrast to 54.5% of the Rwandese who were farmers before displacement and 61.7% who had become farmers since their displacement. These findings are mirrored in the fact that less than 1% of the Somalis rely on agriculture as a source of food.

While 46.5% of the Somalis and 19.6% of the Rwandese had no occupation before displacement, a hefty 79.8% of the Somalis and only 19.6% of the Rwandese claimed no occupation since displacement. Differences between national groups in occupation after displacement were also significant; χ^2 ^= 657.8, p < .000.

### Nutrition

Of the total number of respondents, 93.9% cited the food-aid provided by the UNHCR as their primary source of food while barter trade was an important food source for 3.1% and agriculture for 2.2%. Notably, 99% of the Somalis depended on food-aid. Barter trade and agriculture combined were a primary food source for at least 8% of the Rwandese. The mean number of meals was 1.43 (SD = 0.53). Only 71 people (5%) of the sample had fish or meat as part of their diet.

### Economic Indicators

Of the sample, 1251 (88.2%) have a rent-free accommodation (semi-permanent house). The asset value used in analyses does not include the value of rent-free accommodation, a free water supply (though not piped), subsidized educational opportunities for primary school children, subsidised health care and free recreational sports access. The mean asset value was $ 9.99 (SD = 12.1).

### Migration Factors

Migration into Nakivale camp began as early as 1952 and was still going on in 2003. The greatest influx were in 1991 (n = 357), and 1994 (n = 786) which coincide with the Somali war and the Rwanda genocide and respectively. The mean number of years spent in the camp was 3.88 (SD = 2.64). Everyone had been displaced at least once.

#### Mental Health Indicators

The mean number of separate traumatic events experienced over the lifetime was 9.98 (SD = 5.68). Over the past year, a mean of 0.29 (SD = 1.27) events were experienced. The mean sum score on the PDS (number of separate PTSD symptoms) was 14.1 (SD = 13.5) from a possible 51. The mean scores on the symptom sub-clusters were: arousal M = 3.76 (SD = 4.2), intrusion (M = 5.0 (SD = 4.85) and avoidance M = 5.32 (SD = 5.61). The mean score on depression on the HSCL-25 was 0.77 (SD = 0.81) and 0.75 (SD = 0.74) on anxiety. The mean number of separate physical complaints in the past month was 4.35 (SD = 2.54) and 2.07 (SD = 2.26) functioning deficits within the same period.

### Nationality Differences

The two national groups were clearly distinct in general characteristics: The Somalis tended to have larger households than the Rwandese, had spent more years in the camp, had fewer meals daily but were a younger population and had had more years of education. Differences in education did not however translate into differences in value of possessions, which were insignificant across nationality and gender (M = $9.99, SD = 12.1).

The Somalis had experienced more lifetime traumatic events than the Rwandese, more traumatic events within the past year and therefore scored higher on the PDS. The Somalis also scored higher than the Rwandese on separate PTSD symptom clusters: intrusions, avoidance, arousal, active avoidance, passive avoidance, anxiety symptoms, and depression symptoms.

The different levels of trauma exposure and PTSD prevalence did not occasion any nationality differences in reported number of health complaints or in functioning deficits.

The Somali national group was more homogeneous than the Rwandese national group. For example, within the Somali national group, there were no differences in PTSD prevalence, number of lifetime and recent event types, PDS sum score, intrusive PTSD symptoms, passive and active avoidance PTSD symptoms, depression scores, number of health or functioning deficits, number of years spent in the camp, age or value of possessions between Somali men and women. However, the women scored higher on arousal symptoms of PTSD and anxiety symptoms than the men. Somali women also had fewer meals, had had fewer years of schooling, had larger households than Somali men and reported less substance use (khat).

Among the Rwandese, the men had a higher PTSD prevalence, a higher number of lifetime traumatic events, a higher PDS sum score, higher active avoidance and passive avoidance symptom scores, and higher depression scores. Rwandese men also scored higher on intrusion symptoms as well as avoidance symptoms taken as a whole and had less to eat than the women. They also reported more functioning deficits than the women and more substance use.

The Rwandese women had larger households, however, had fewer possessions, had had fewer years of schooling and were younger than the men.

Gender differences in recent traumatic events, arousal PTSD symptoms, anxiety levels, health deficits and years spent in the camp among the Rwandese were not significant.

### Gender

Gender did not prove to be a uniform factor across cultures. While the Rwandese women had the fewest number of lifetime traumatic events, the lowest prevalence of PTSD as well as the lowest PDS sum score, the Somali women were highly traumatised, had as many events as the Somali men and as high a PDS score. On all indicators of ill health, the Somali women scored higher than the Rwandese women. Somali women had experienced more lifetime and recent traumatic events than Rwandese women, and therefore scored higher on the PDS and on all three symptom clusters. Somali women also scored higher than Rwandese women on the avoidance sub-clusters (active and passive avoidance) as well on anxiety and depression symptoms

The Somali women had spent more years in the camp than the Rwandese women, had larger households and less to eat. Rwandese women reported more substance use (crude liquor) than Somali women.

There were no differences in age, level of education, value of possessions or health and functioning between the two national groups of women.

Among the men, differences were also evident along nationality lines. Somali men scored higher than Rwandese men on all ill-health parameters: they had experienced more lifetime traumatic events and scored higher on the PDS. Somali men also displayed a higher number of intrusive, avoidance and arousal symptoms than Rwandese men. Somali men scored higher on the avoidance sub-clusters (active and passive avoidance) as well as on depression symptoms than Rwandese men.

Somali men also had spent more years in the camp, had larger households and had less to eat than the Rwandese. They were also younger and better educated. Rwandese men reported the highest use of addictive substances (in this case local alcoholic brew). The differences between number of recent events experienced by Somali and Rwandese men, health and functioning were not significant. *(A table summarising means of important variables across gender and nationality is provided at the end of the manuscript: see *Table 1. *A table showing t-tests for variable differences across gender and nationality is attached as an Additional file: see *Additional file 1)

**Table 1:** Means of important indicators across national and gender groups (Standard Deviation in brackets)

Key Indicators	Camp	Men	Women	Somali	Rwandese	Somali Men	Somali Women	Rwandese Men	Rwandese Women
No. of Events (Lifetime)	9.98 (5.68)	11 (5.54)	9.17 (5.66)	11.95 (6.17)	8.86 (5.05)	11.75 (5.97)	12.16 (6.38)	10.47 (5.16)	7.77 (4.67)

No. of recent Events	0.29 (1.27)	0.3 (0.99)	0.29 (1.45)	0.45 (1.93)	0.2 (0.63)	0.38 (1.3)	0.53 (2.42)	0.23 (0.71)	0.17 (0.58)

PDS Sum Score	14.1 (13.54)	14.77 (13.33)	13.53 (13.69)	21.17 (16.19)	10.05 (9.7)	19.81 (15.82)	22.58 (16.48)	11.17 (9.77)	9.28 (9.58)

Arousal symptoms	3.76 (4.2)	3.77 (4.03)	3.75 (4.34)	5.73 (5.23)	2.63 (2.95)	5.19 (4.89)	6.28 (5.52)	2.75 (2.89)	2.56 (2.99)

Intrusion symptoms	5.0 (4.85)	5.22 (4.9)	4.83 (4.81)	7.19 (5.98)	3.76 (3.51)	6.71 (6.06)	7.7 (5.89)	4.15 (3.51)	3.49 (3.49)

Avoidance symptoms	5.32 (5.61)	5.78 (5.72)	4.95 (5.5)	8.25 (6.46)	3.66 (4.25)	7.91 (6.62)	8.6 (6.28)	4.27 (4.42)	3.23 (4.09)

Active Avoidance	2.79 (2.82)	2.97 (2.79)	2.65 (2.83)	4.46 (2.95)	1.84 (2.24)	4.27 (2.93)	4.65 (2.97)	2.05 (2.27)	1.7 (2.21)

Passive Avoidance	2.53 (3.35)	2.81 (3.54)	2.3 (3.16)	3.8 (4.28)	1.81 (2.39)	3.65 (4.47)	3.95 (4.08)	2.22 (2.54)	1.53 (2.24)

Anxiety	0.75 (0.74)	0.72 (0.70)	0.78 (0.77)	0.95 (0.91)	0.64 (0.59)	0.81 (0.82)	1.09 (0.98)	0.65 (0.58)	0.63 (0.59)

Depression	0.77 (0.81)	0.81 (0.81)	0.73 (0.81)	1.33 (1.01)	0.44 (0.42)	1.25 (0.98)	1.40 (1.03)	0.49 (0.44)	0.42 (0.41)

Meals	1.43 (0.53)	1.39 (0.51)	1.46 (0.54)	1.06 (0.34)	1.64 (0.5)	1.10 (0.38)	1.02 (0.29)	1.60 (0.49)	1.67 (0.5)

Health complaints sum score	4.35 (2.54)	4.18 (2.57)	4.48 (2.52)	4.22 (2.77)	4.42 (2.41)	4.05 (2.65)	4.41 (2.87)	4.28 (2.50)	4.5 (2.34)

Functioning Deficits	2.07 (2.26)	2.44 (2.36)	1.81 (2.16)	2.45 (3.14)	2.02 (2.09)	2.23 (3.01)	2.71 (3.31)	2.48 (2.21)	1.71 (1.97)

Drug sum score	0.74 (3.25)	1.33 (4.65)	0.27 (1.22)	0.27 (1.57)	0.99 (3.87)	0.53 (2.18)	0.004 (0.06)	1.89 (5.72)	0.4 (1.46)

Household size	5.39 (3.27)	5.05 (3.26)	5.66 (3.25)	6.68 (3.76)	4.65 (2.69)	6.19 (3.44)	7.19 (3.99)	4.23 (2.85)	4.93 (2.53)

Years spent in camp	3.88 (2.64)	4.12 (2.67)	3.69 (2.60)	5.7 (2.03)	2.85 (2.38)	5.74 (2.14)	5.65 (1.91)	2.96 (2.39)	2.78 (2.37)

Education	3.82 (3.83)	4.92 (4.18)	2.95 (3.28)	5.04 (4.49)	3.13 (3.20)	6.83 (4.45)	3.2 (3.73)	3.57 (3.38)	2.84 (3.04)

Asset value ($)	9.99 (12.1)	11.59 $(14)	8.73 (10.15)	(10.21) (12.24)	9.87 (12.0)	11.04 (12.5)	9.36 (11.9)	11.98 (15.0)	8.43 (9.20)

Age	31.65 (12.7)	32.46 (13.1)	31.00 (12.4)	29.55 (12.3)	32.84 (12.8)	28.89 (11.2)	30.22 (13.4)	34.98 (13.8	31.37 (11.9)

Sexual Events	0.79 (1.34)	0.43 (0.92)	0.95 (1.47)	1.29 (1.87)	0.64 (1.11)	0.00 (0.00)	1.36 (1.88)	0.45 (0.94)	0.77 (1.19)

Violent Events	6.23 (3.25)	6.69 (3.12)	5.84 (3.27)	6.93 (3.31)	5.83 (3.12)	6.99 (3.16)	6.89 (3.54)	6.52 (3.01)	5.36 (3.05)

#### Prevalence

The prevalence of PTSD in the whole sample was 37.8% (n = 538). Gender and nationality differences were evident, with more men (42.7%, n = 269) suffering than women (34%, n = 269) and more Somali (48.1%, n = 248) than Rwandese (32%, n = 290). Within nationality groups, further differences manifested themselves. While Somali men and women suffered equally (48.1%, n = 126; 48%, n = 122) respectively, Rwandese men suffered more from PTSD than the women (38.9%, n = 143; 27.3%, n = 147). *(A table summarising the PTSD prevalence rates is included at the end of the manuscript: see *Table 2).

**Table 2:** PTSD prevalence according to gender and nationality

	Total	Men	Women
Camp (n = 538)	37.8%	42.7%	34%

Somali (n = 248)	48.1%	48.1%	48.1%

Rwandese (n = 290)	32%	38.9%	27.3%

Nationality differences in PTSD prevalence were significant: χ^2 ^(df = 1) = 36.02; p < .000. Gender differences in PTSD prevalence were significant only within the Rwandese national group: χ^2 ^(df = 1) = 13.52; p < .000. Gender differences in PTSD prevalence across cultural groups were also significant: Somali women had a higher PTSD prevalence than Rwandese women: χ^2 ^(df = 1) = 33.08; p < .000, while Somali men showed a higher prevalence of PTSD than Rwandese men: χ^2 ^= 5.27; p = .022.

### Event types

The single most reported event was witnessing dead or mutilated bodies, reported by 73.5% of the respondents (n = 1065). Other often-reported events were shelling or bomb attack, reported by 69.3%; witnessed injury with a weapon, reported by 67.7%; experiencing crossfire or sniper attacks, reported by 60.3% and experiencing burning houses, reported by 60.2%.

Other common traumatic events included witnessing beatings or torture (59.1%), witnessing combat (50.9%), witnessing killing or murder (50.9%) and harassment by armed personnel (48.7%). The percentages overlap as most respondents experienced multiple traumatic events.

Sexual crimes appear to have been less important than violent crimes in this population. Rape was reported by 4.2% of the respondents (both Somali and Rwandese), sexual harassment by 6.0%, forced prostitution by 2.1% (mainly Rwandese), forced circumcision by 4.1% (mainly Somalis) and sex for food or security by 1.4%. Many more had witnessed the same events happen to someone else, however: rape (14.1%), forced prostitution (12.7%) and forced circumcision (9.8%).

Of the 34 traumatic events on the Event List, 10 events involved sexual violence. The mean number of sexual violence events reported was 0.79 (SD = 1.34), compared to the mean number of the ten most reported violent events, 6.23 (SD = 3.25). Somali women reported the highest number of sexually violent events (1.36, SD = 1.88), although this was less than the number of violent events they reported (M = 6.89, SD = 3.54). Somali men reported the least number of sexually violent events (M = 0.00, SD = 0.00), although they reported a high number of violent events (M = 6.99, SD = 3.16). Somali women reported a significantly higher number of traumatic sexually violent events than Somali men (t(246) = 11.33; p < .000), and than Rwandese women (t(339) = 4.5; p < .000), who also reported a higher number of violent traumatic events (M = 5.36, SD = 3.05) than sexually violent events (M = 0.77, SD = 1.19).

**Figure 1:**
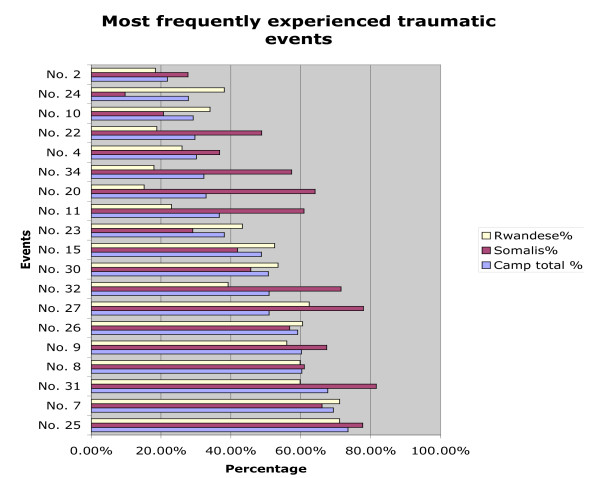
**illustrates the occurrence of lifetime traumatic events by nationality**.

**Figure 2:**
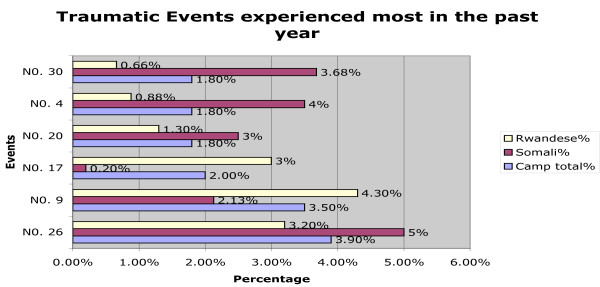
**depicts the occurrence of recent (within the past year) traumatic events by nationality**.

Rwandese men also reported more non-sexual violent traumatic events (6.52, SD = 3.01) than sexually violent events (M = 0.45, SD = 0.94). Rwandese women had experienced significantly more sexually violent events (t(846) = 4.46; p < .000) than the men. The difference in sexually violent events reported by Somali and Rwandese men did not reach significance. *(A figure illustrating occurrence of lifetime traumatic events is included at the end of the manuscript: see *Figure 1. *A second figure depicting recent (within the past year*) *traumatic events is included as *Figure 2). *The Event List is included as a table at the end of the manuscript: see *Table 3).

**Table 3:** Prevalence (%) of traumatic event types experienced ever (lifetime) and in the past year by nationality

	**Ever**			**In the past year**		
Events n (%)	Camp Total	Somali	Rwande	Camp total	Somali	Rwande

1. Abduction or forceful recruitment	124 (8.7)	112	12	3 (0.2)	3 (0.2)	0

2. Accident	310 (21.8)	143 (27.7)	167 (18.4)	14 (1.0)	7	7

3. Beating by spouse (*for women)	111 (7.8)	18	93	19 (1.3)	2	17

4. Beating or torture	428 (30.1)	190 (36.8)	238 (26)	26 (1.8)	18 (3.5)	8 (0.1)

5. Child marriage	58 (4.1)	23	35	0	0	0

6. Combat situation	33 (2.3)	4	29	1 (0.1)	0	1

7. Shelling/bomb attack	985 (69.3)	341 (66.1)	644 (71.1)	16 (1.1)	4	12

8. Experienced crossfire or sniper attack	857 (60.3)	315 (61)	542 (59.8)	9 (0.6)	5	4

9. Experienced burning houses	856 (60.2)	348 (67.4)	508 (56)	50 (3.5)	11 (2.1)	39 (4.3)

10. Property confiscated by officials	415 (29.2)	107 (20.7)	308 (34)	7 (0.5)	3	4

11. Dangerous evacuation	522 (36.7)	314 (60.9)	208 (23)	5 (0.4)	5	0

12. Injured with weapon	230 (16.2)	156	74	4 (0.3)	4	0

13. Forced circumcision *(for women)	59 (4.1)	50	9	1 (0.1)	1	0

14. Forced prostitution or sexual slavery	30 (2.1)	3	27	0	0	0

15. Harrassed by armed personnel	692 (48.7)	216 (41.9)	476 (52.5)	19 (1.3)	14	5

16. Imprisoned	221 (15.5)	65	156	15 (1.1)	9	6

17. Experienced poisoning or witchcraft	246 (17.3)	12	234	28 (2.0)	1 (0.2)	27 (3)

18. Rape	60 (4.2)	34	26	7 (0.5)	1	6

19. Sexual harassment (touch)	85 (6.0)	29	56	10 (0.7)	5	5

20. Experienced robbery or looting	468 (32.9)	331 (64.1)	137 (15.1)	25 (1.8)	13 (2.5)	12 (1.3)

21. Sex for food/security	20 (1.4)	6	14	0	0	0

22. Witnessed abduction or forced recruitment	422 (29.7)	252 (48.8)	170 (18.8)	5 (0.4)	4	1

23. Witnessed accident	542 (38.1)	150 (29.1)	392 (43.3)	20 (1.4)	6	14

24. Witnessed suicide	395 (27.8)	50 (9.7)	345 (38.1)	13 (0.9)	10	3

25. Witnessed dead or mutilated bodies	1045 (73.5)	401(77.7)	644(71.1)	16 (1.1)	11	5

26. Witnessed beatings or torture	841 (59.1)	293 (56.8)	548 (60.5)	55 (3.9)	26 (5)	29 (3.2)

27. Witnessed combat s	724 (50.9)	402 (77.9)	322 (62.4)	8 (0.6)	8	0

28. Witnessed forced circumcision	139 (9.8)	116	23	15 (1.1)	14	1

29. Witnessed forced prostitution	180 (12.7)	56	124	10 (0.7)	8	2

30. Witnessed harassment by armed personnel	721 (50.7)	236 (45.7)	485 (53.5)	25 (1.8)	19 (3.7) 6	(0.7)

31. Witnessed injury with weapon	963 (67.7)	421 (81.6)	542 (59.8)	13 (0.9)	9	4

32. Witnessed killing or murder	724 (50.9)	369 (71.5)	355 (39.2)	7 (0.5)	6	1

33. Witnessed rape	201 (14.1)	106	95	12 (0.8)	6	6

34. Witnessed robbery/looting	459 (32.3)	296 (57.4)	163 (18)	11 (0.8)	8	3

As a measure of the internal validity of the data and the diagnoses, correlations (Pearson) were carried out between key indicators. For example, the PDS sum score, referring to the total number of PTSD symptoms, correlates significantly with the sum of arousal symptoms (0.912); the sum of avoidance symptoms (0.933); the sum of intrusion symptoms (0.922) as well as with the sum of functioning deficits (0.657). It also correlates significantly with the sum of anxiety symptoms (0.784), the sum of depression symptoms (0.858) and the total number of traumatic events (0.544).

It does not, however, correlate significantly with the amount of addictive substances consumed (the drug sum score) and only weakly with the sum of physical health deficits, suggesting that physical ill-health in this population is not predicted by mental ill-health alone.

In turn, the anxiety sum score correlates significantly with the PTSD arousal (0.785), avoidance (0.699) and intrusion symptoms (0.697) and functioning loss (0.640). The sum of depression symptoms also correlates significantly with functioning loss (0.645), arousal (0.829), avoidance (0.786) and intrusion symptoms (0.766).

(*Correlations of key indicators are summarised as a table: see *Table 4).

**Table 4:** Pearson's Correlation between key health variables.

Indicator	Statistic	Func sum	Drug sum	PDS sum	Arou sum	Avoi sum	Intru sum	Hscl anxs	Hscl deps	No. of events	Heal sum
Function Sum score	Pear.Cor	1	.064	.657	.583	.625	.592	.640	645	.455	.232
	Sig(2)	.	.133	.000	.000	.000	.000	.000	.000	.000	.000
	N	560	550	560	560	560	560	555	555	555	560

Drug Sum score	Pear.Cor	.064	1	.022	.007	.023	.028	.002	-.027	.049	-.034
	Sig.(2)	.133	.	.408	.790	.380	.239	.950	.319	.068	.002
	N	550	1401	1401	1401	1401	1401	1393	1393	1395	1401

PDS Sum score	Pear.Cor	.657	.022	1	.912	.933	.922	.784	.858	.544	.362
	Sig.(2)	.000	.408	.	.000	.000	.000	.000	.000	.000	.000
	N	560	1401	1421	1421	1421	1421	1413	1413	1414	1421

Arousal Sum score	Pear.Cor	.583	.007	.912	1	.776	.781	.785	.829	.503	.366
	Sig.(2)	.000	.790	.000	.	.000	.000	.000	.000	.000	.000
	N	560	1401	1421	1421	1421	1421	1413	1413	1414	1421

Avoidance Sum score	Pear.Cor	.625	.023	.933	.776	1	.774	.699	.786	.476	.315
	Sig (2)	.000	.380	.000	.000	.	.000	.000	.000	.000	.000
	N	560	1401	1421	1421	1421	1421	1413	1413	1414	1421

Intrusion Sum score	Pear.Cor	.592	.028	.922	.781	.774	1	.697	.766	.532	.329
	Sig.(2)	.000	.289	.000	.000	.000	.	.000	.000	.000	.000
	N	560	1401	1421	1421	1421	1421	1413	1413	1414	1421

HSCL Anxiety Sum score	Pear.Cor	.640	.002	.784	.785	.699	.697	1	.776	.511	.467
	Sig.(2)	.000	.950	.000	.000	.000	.000	.	.000	.000	.000
	N	555	1393	1413	1413	1413	1413	1413	1413	1406	1413

HSCL Depression Sum score	Pear.Cor	.645	-.027	.858	.829	.786	.766	.776	1	.526	.333
	Sig.(2)	.000	.319	.000	.000	.000	.000	.000	.	.000	.000
	N	555	1393	1413	1413	1413	1413	1413	1413	1406	1413

Total No. of Event types	Pear.Cor	.455	.049	.544	.503	.476	.532	.511	.526	1	.254
	Sig.(2)	.000	.068	.000	.000	.000	.000	.000	.000	.	.000
	N	555	1395	1414	1414	1414	1414	1406	1406	1414	1414

Health Sum score	Pear.Cor	.232	-.034	.362	.366	.315	.329	.467	.333	.254	1
	Sig. (2)	.000	.200	.000	.000	.000	.000	.000	.000	.000	.
	N	560	1401	1421	1421	1421	1421	1413	1413	1414	1422

Correlation is significant at the 0.01 level (2-tailed).

## Discussion

This refugee population is very poor, with individual possessions totalling less than ten dollars in worth. It is also under-nourished, with individuals eating little over one meal a day, containing no fish or meat. It is also a population with little education and therefore few employment prospects.

The refugees are also physically unhealthy, reporting at least four separate physical complaints each within a one-month period. This could be attributed partly to poor nutrition, and partly to mental ill health, which often manifests itself in psychosomatic symptoms.

It is conceivable that this is the profile of many refugee populations in Africa. The value of this information is evident because conflicts continue to proliferate in Africa and even more people are forced to migrate. For example, Uganda is receiving an influx of thousands of new refugees from the Congo. Such information is vital for planning emergency and other services in the host countries [16].

The refugees had spent an average of over three years in the refugee settlement, a place that did not guarantee absolute safety.

The sample manifested a high traumatic load, with over nine separate traumatic events each, including sexual events. This is reflected in the high PTSD symptom load of 14 separate symptoms. The separate PTSD symptom clusters, as well as anxiety and depression symptoms were also high across the board. This contributed to the physically run-down state of the refugees as well as to functioning deficits. The refugees reported at least two such deficits on the average. Such deficits include the inability to engage in economically productive activities – which further complicates an already precarious economic situation – as well as the inability to benefit from educational opportunities. Other functioning deficits include dysfunctional marital and family life, and addictive substance abuse.

The rates of prevalence of PTSD within this refugee population are consistent with findings from other post-conflict populations. Notably, the prevalence rates among the Somali respondents were exceptionally high (half of the population). The Somalis have experienced more traumatic events and are more vulnerable across all mental health and nutrition variables, which could predispose them to mental illness.

It is possible that this is an especially vulnerable sample of the Somali refugees, possibly a self-select group that could have been exposed to traumatic events of unusual number and severity during numerous conflict situations and a prolonged forced migration process. However, it is also conceivable that Somali war survivors in general have had an unusually high exposure to conflict-related events during the long periods of repeated unrest. The Somali population was also more vulnerable to recent traumatic events that took place within the refugee settlement (in the past year). That such events continue to take place shows that such refuges do not offer absolute safety for refugees.

The researchers did not set out to establish construct validity of the PTSD construct within these cultural groups. However, the significant correlations between various health measures such as posttraumatic stress disorder symptom sum score, depression and anxiety scores and subsequent functioning deficits may indicate the validity of the PTSD construct in these populations (*see *Table 4). The researchers did seek to establish criterion validity and, as mentioned before, the results are reported fully in [35]. Notably, the HSCL-25 in both languages had a low Kappa score (0.31 when taken together). This indicates a deficiency in the accuracy of the rendition of the HSCL-25 into the local languages Kinyarwanda and Somali, thus limiting the reliability of the depression and anxiety measures.

## Conclusion

As shown, post-conflict refugee populations relocated to low-income countries in Africa may have high prevalence of both PTSD and depression, which may persist years after the causative experiences. It is therefore clear that allocations for mental health provision are just as urgent as any emergency material provisions made for forced migrants – maybe even more so. This is also very important information for third host countries, for example the high-income countries to which the entire Somali sample in this study was later resettled.

Provision of access to mental health interventions should be taken into account by all agencies that offer relief to refugees and other migrant populations. As noted, mental illness severely handicaps the functioning of sufferers across a range of domains, reducing their capacity to reconstruct their lives or build up progressive communities. Mental ill health also contributes to poor physical health. As such, untreated mental illness carries an enormous hidden economic cost that may hinder the recovery of forced migrant populations. The identification and treatment of different mental illnesses such as posttraumatic stress disorder and depression among post-conflict populations is therefore a matter of urgency.

This group of researchers initiated a mental health intervention program based on these findings. The main aim of the program was to build capacity among local lay interviewers and therapists, with a view to providing treatment for posttraumatic stress disorder and depression[36].

### Limitations

Although no effort was spared to make this study as comprehensive as possible, the study manifests some limitations. Firstly, the CIDI was used as a criterion for validation of the PDS in the local languages. However, the CIDI had not been validated for this setting, and it is not exclusively a clinician-administered diagnostic interview, thus limiting its validity as a criterion.

Secondly, it would have been informative to have data from the host Ugandan population – for example regarding income or physical and mental health indicators – for purposes of comparison, which is not the case. This could be improved on in future studies.

## Competing interests

The authors declare that they have no competing interests.

## Authors' contributions

LPO drafted the manuscript, managed and participated in the data collection and was involved in data analysis and interpretation.

TE and FN conceived and designed the study, participated in data collection, guided data analysis and interpretation and critically revised the document before submission. VE participated in data collection and was involved in data management, analysis and interpretation.

ES and MO participated in data collection and interpretation.

## Supplementary Material

Additional file 1 **Table showing independent t-test values across gender and nationality for key indicators**. The data provided represents the statistical analysis of t-test values for various variables across gender and nationality. Click here for file
