# Supplementary material for: Trauma, poverty and mental health among Somali and Rwandese refugees living in an African refugee settlement – an epidemiological study
Source: Confl Health. 2009 May 26;3:6. doi: 10.1186/1752-1505-3-6 (PMC2695430; doi:10.1186/1752-1505-3-6)
Supplement: Additional file 1 — Table showing independent t-test values across gender and nationality for key indicators. The data provided represents the statistical analysis of t-test values for various variables across gender and nationality. [file 1752-1505-3-6-S1.pdf]

**Additional File 1** *Table showing independent t-test values across gender and nationality for key indicators*

| Indicator | Men S/R | Women S/R | Somali M/F | Rwandese M/F | Nationality S/R |
|---|---|---|---|---|---|
| **No. of lifetime events** | T(501) = 2.79 p < .005 | T(383) = 9.78 p < .000 | ns | T(901) = 8.18 p < .000 | T(896) = 9.65 p <.000 |
| **No. of recent events** | ns | T(265) = 2.28 p = .023 | ns | ns | T(572) = 2.9 p < .004 |
| **PDS sum score** | T(400) = 7.84 p < .000 | T(334) = 11.92 P < .000 | ns | T(904) = 2.89 p < .004 | T(727) = 14.21 p <. .000 |
| **Intrusions** | T(385) = 6.14 p < .000 | T(338) = 10.54 p < .000 | ns | T(904) = 2.8 p < .005 | T(719) = 11.91 p < .000 |
| **Avoidance** | T(422) = 7.75 p < .000 | T(355) = 12.41 p < .000 | ns | T(746) = 3.58 p < .000 | T(772) = 14.45 p < .000 |
| **Active Avoidance** | T(471) = 10.27 p < .000 | T(388) = 14.08 p< .000 | ns | T(903) = 2.26 p= .024 | T(853) = 17.45 p < .000 |
| **Passive Avoidance** | T(378) = 4.66 p < .000 | T(325) = 8.83 p < .000 | ns | T(722) = 4.2 p < .000 | T(698) = 9.69 p < .000 |
| **Arousal** | T(389) = 7.26 p < .000 | T(323) = 10.06 p< .000 | T(500) = 2.36 p = .019 | ns | T(703) = 12.35 p < .000 |
| **Anxiety** | ns | T(339) = 6.91 P <.000 | T(490) = 3.55 p < .000 | ns | T(755) = 6.88 p < .000 |
| **Depression** | T(331) = 11.74 p < .000 | T(289) = 14.77 p < .000 | ns | T(742) = 2.52 p = .012 | T(614) = 18.93 p < .000 |
| **Household size** | T(494) = 7.55 p < .000 | T(352) = 8.22 p < .000 | T(514) = 3.03 p < .003 | T(725) = 3.8 p < .000 | T(819 = 10.81 p < .000 |
| **Camp years** | T(626) = 15.02 p < .000 | T(784) = 16.83 p < .000 | ns | ns | T(1203) = 23.4 p < .000 |
| **Education** | T(454) = 9.95 p < .000 | ns | T(496) = 9.99 p < .000 | T(902) = 3.38 p < .001 | T(805) = 8.45 P < .000 |
| **Asset value** | ns | ns | ns | T(555) = 4.04 | ns |

| | | | | | |
|---|---|---|---|---|---|
| | | | | p < .000 | |
| **Meals** | T(622) = 14.66 p < .000 | T(755) = 23.0 p < .000 | T(486) = 2.58 p = .010 | T(902) = 2.03 p = .043 | T(1371) = 26.24 p < .000 |
| **Age** | T(625) = 5.89 p < .000 | ns | ns | T(898) = 4.19 p < .000 | T(1412) = 4.71 p < .000 |
| **Drug Sum** | T(494) = 4.1 p< .000 | T(539) = 6.22 p < .000 | T(253) = 3.85 p < .000 | T (391) = 4.84 p < .000 | T(1299) = 4.95 p < .000 |
| **Functioning** | ns | ns | ns | T (377) = 3.94 p < .000 | ns |
| **Health** | ns | ns | ns | ns | ns |
| **Sexual Events** | ns | T(339) = 4.5 p < .000 | T(246) = 11.33 p < .000 | T(846) = 4.46 p < .000 | T(316) = 5.3 p < .000 |
